# ﻿*Ophiorrhizaliuyanii* (Rubiaceae), a new species from south-western China and northern Vietnam

**DOI:** 10.3897/phytokeys.248.135078

**Published:** 2024-10-30

**Authors:** Chu-Yu Liu, Xiao-Wen Liao, Li-Chun Ye, Yun-Hong Tan, Khang Sinh Nguyen, Tran Duc Thien, Lei Wu

**Affiliations:** 1 School of Minerals Processing and Bioengineering, Central South University, Changsha 410083, China Central South University Changsha China; 2 College of Forestry, Central South University of Forestry and Technology, Changsha 410004, China Central South University of Forestry and Technology Changsha China; 3 Center for Integrative Conservation, Xishuangbanna Tropical Botanical Garden, Chinese Academy of Sciences, Mengla 666303, China Xishuangbanna Tropical Botanical Garden, Chinese Academy of Sciences Mengla China; 4 Institute of Ecology and Biological Resources, Vietnam Academy of Science and Technology, 18, Hoang Quoc Viet Road, Cau Giay, Hanoi 10072, Vietnam Graduate University of Science and Technology, Vietnam Academy of Science and Technology Hanoi Vietnam; 5 Graduate University of Science and Technology, Vietnam Academy of Science and Technology, 18 Hoang Quoc Viet, Cau Giay, Hanoi 10072, Vietnam Institute of Ecology and Biological Resources, Vietnam Academy of Science and Technology Hanoi Vietnam; 6 Regional Research and Development Institute, Ministry of Science and Technology, No. 70, Tran Hung Dao, Hoan Kiem District, Hanoi, Vietnam Regional Research and Development Institute, Ministry of Science and Technology Hanoi Vietnam

**Keywords:** New taxon, *Ophiorrhiza*, Rubiaceae, taxonomy

## Abstract

*Ophiorrhizaliuyanii*, a new species from south-western China and northern Vietnam, is described and illustrated. The new species is characterised by the glabrous surfaces on almost all plant parts, congested inflorescences and broad-ovate to ovate bracts 9–22 × 4–11 mm. It morphologically differs from the closest species, *O.baviensis*, mainly in most plant parts being glabrous, bracts broad-ovate to ovate, apex acute or sometimes obtuse, corollas inside with a ring of white hairs at the middle and anthers inserted near the middle in long-styled flowers. According to IUCN Categories and Criteria, *O.liuyanii* is assessed as Least Concern (LC).

## ﻿Introduction

The genus *Ophiorrhiza* Linnaeus is an Indo-Malesian genus of Rubiaceae with species distributed in tropical and subtropical regions of Asia, with only a few extending to Australia, New Guinea and the Pacific Ocean (Tran 2005; Chen and Taylor 2011; Hareesh et al. 2015; Li 2020; Schanzer and Nabatov 2022; Shang et al. 2024). Representatives of this genus are annual or perennial herbs or rarely sub-shrubs, easily recognised by having obcordate and compressed fruits that are dehiscent with two valves along a transverse slit at the top (Darwin 1976; Lo 1990; Wu et al. 2015) and usually growing in moist locations or stream-sides under evergreen forests (Darwin 1976; Deb and Mondal 1997; Chen and Taylor 2011; Hareesh et al. 2015). In spite of the clear monophyly of the whole genus, based on capsule shape, the total species number of the genus is unclear and is estimated to be from 200 species (Lo 1999; Li 2020) to as many as 300 species (Alfeche et al. 2020; Taher et al. 2020; Zhou et al. 2020; Idrees et al. 2023) due to the lack of a worldwide revision.

China is a diversity centre of *Ophiorrhiza* with about 74 taxa recorded (Wu et al. 2017, 2018; Tu et al. 2018; Yang et al. 2018; Hu et al. 2021; Liu et al. 2023; Shang et al. 2024; Zhan et al. 2024). Most of Chinese *Ophiorrhiza* are distributed in southern and south-western China, particularly in Guangxi and Yunnan Provinces (Lo 1999; Chen and Taylor 2011). While examining *Ophiorrhiza* specimens at PE Herbarium in 2013, we found an unusual sheet with congested inflorescences, broadly ovate bracts and winged corolla outside. Due to the single specimen and lack of information inside the corolla, we tentatively treated it as *O.baviensis* Drake and thought that the difference in bracts might be a variable character within this species. In recent field surveys in Menghai County, south-western Yunnan, the peculiar plants of this species with fruits in 2014 and flowers in 2024 were observed and re-collected. After carefully examining fresh and dried material of the abovementioned species, we found that it is distinctly different from *O.baviensis* by the glabrous surfaces on most plant parts, larger bracts broad-ovate to ovate, the indumentum inside corollas and the placement of stigma and anthers (Wu et al. 2019). Further, from a comprehensive comparison between this peculiar plant with other known species of the genus, we concluded that it represents a new taxon, which is described hereafter.

## ﻿Materials and methods

Field observations were carried out in south-western China in 2014 and 2024 and northern Vietnam in 2022. The morphological characteristics of a new *Ophiorrhiza* species were observed and measured in the field and laboratory. The morphological variations of 30 individuals were measured with a ruler and a micrometer. Specimens of the new species were preserved in the Forest Plant Herbarium (CSFI) of Central South University of Forestry and Technology and other herbaria (BNU, CSFI, HITBC, HN and LE). Acronyms for all herbaria in the text follow Thiers (2024). The conservation status of the new species was evaluated, based on field observations and referred to the IUCN Red List Guidelines (IUCN 2023).

## ﻿Taxonomic treatment

### ﻿ Ophiorrhiza liuyanii

Taxon classificationPlantaeGentianalesRubiaceae

L.Wu, Y.H.Tan & K.S.Nguyen sp. nov.

5E448AFD-95DA-5C9E-9E83-FADEBEBC4DF6

urn:lsid:ipni.org:names:77351093-1

Figs 1 , 2A–K

#### Type.

China • Yunnan Province: Menghai County, Mengsong Village, growing along a stream or on moist slopes under densely evergreen broad-leaved forests, 21°30'37.36"N, 100°30'17.33"E, elevation 1715 m, 13 Apr 2024 (fl.), *X.W. Liao LXW0217* (holotype: CSFI!; isotypes: CSFI!).

#### Diagnosis.

Morphologically similar to *O.alatiflora* and *O.baviensis*, but the new species differs from the former by its congested (vs. developing) inflorescences and infructescences, broad-ovate to ovate (vs. linear or linear-lanceolate) bracts, 4–11 (vs. 0.8–1.5) mm wide and from the latter by its glabrous (vs. densely pubescent or puberulent) peduncles, broad-ovate to ovate (vs. lanceolate) bracts, corollas tube inside with (vs. without) a ring of white hairs at the middle in long-styled flowers.

#### Description.

Perennial herbs, erect or ascending at the base, up to 80 cm tall; stem, leaves, petiole, stipule, bract, outside flower and capsule glabrous. Leaves generally in equal pairs (usually isophyllous); petioles 1–3 cm, pale green; leaf blades drying papery, dark green adaxially, pale green abaxially, elliptic, oblong or ovate-elliptic, 7–15 × 3–6 cm, cuneate at base, acuminate at apex, margins entire; secondary veins 9–13 at each side; stipules small, broadly triangular, ca. 1 mm long, caducous, with glands at the inner base. Inflorescences congested cymose, many-flowered, drooping at the early stage, then erect; peduncles 1–2 cm long, pale green; bracts broad-ovate to ovate, 9–22 × 4–11 mm, apex acuminate, acute or sometimes obtuse. Flowers heterostylous; pedicels to 3 mm long, puberulent. Calyx puberulent; hypanthium oblate, 1.5–1.8 × 1.8–2.2 mm; lobes triangular to ovate triangular, 0.8–1.6 mm long, acuminate at apex. Corolla white or pinkish-white, subtubular; tube 1.0–1.6 cm long, outside longitudinally winged from apex to base, wings straight or undulate, ca. 0.8–2 mm wide; lobes 5, ovate-triangular, 3.8–4.8 × 2.8–3.5 mm, inside pubescent, apex acute, slightly incurved. Stamens 5; anthers linear, 2.2–3.2 mm long. Stigma bilobed; ovary 2-celled. Long-styled flowers: inside with a ring of white hairs at the middle of the corolla tube and puberulent from the middle up to the throat; stamens included, positioned a little below the middle of the corolla tube; style 8–12 mm long, densely pubescent; stigma positioned at the corolla throat, lobes elliptic, ca. 1.8 mm long. Short-styled flowers: sparsely pubescent at the middle of the corolla tube; stamens reaching slightly beyond corolla throat, not exserted; style 3.8–5.5 mm long, pubescent; stigma lobes lanceolate, ca. 2.8 mm long. Capsules mitriform, ca. 4.5 × 10 mm.

**Figure 1.**
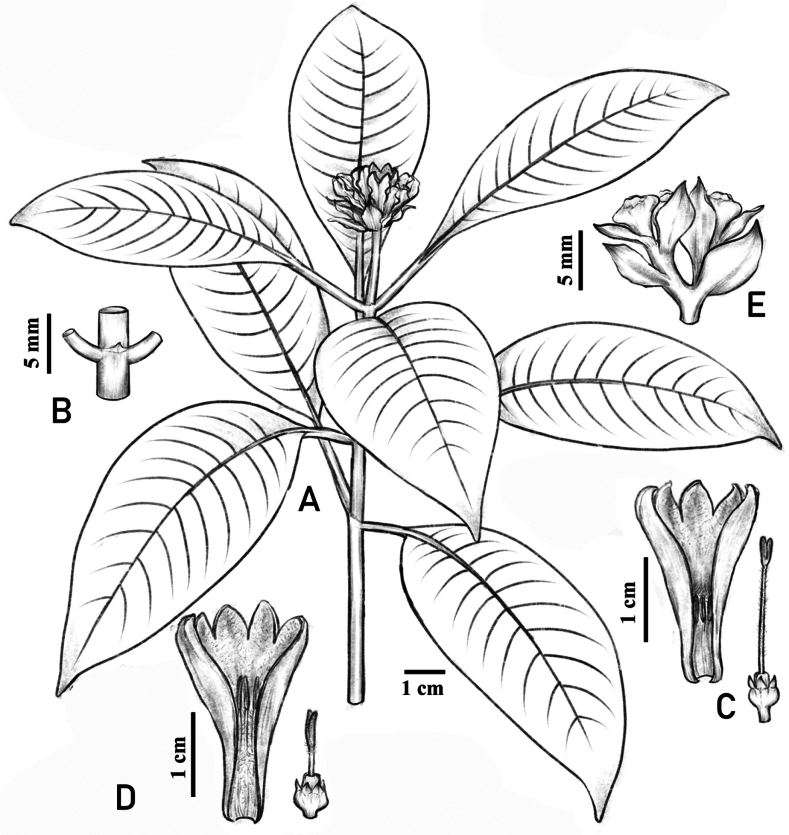
*Ophiorrhizaliuyanii***A** flowering branch **B** stipule **C** longitudinally dissected short-styled flower **D** longitudinally dissected long-styled flower **E** infructescence in side view. Drawn from the holotype by M.M. Cheng.

#### Phenology.

Flowering from April to May; fruiting from May to July.

#### Distribution and habitat.

*Ophiorrhizaliuyanii* is currently known from south-western China (Menghai County of southern Yunnan Province) and north-western Vietnam (Dien Bien Province). It grows along streams or moist places under evergreen broad-leaved forests at an elevation range from 1500–1850 m, in which the vegetation is dominated by the tree families Lauraceae, Fagaceae, Magnoliaceae, Theaceae and Betulaceae, shrub families Ericaceae and Symplocaceae and herbaceous families Urticaceae, Balsaminaceae and Begoniaceae.

#### Preliminary conservation status.

Our field surveys revealed that three populations of *Ophiorrhizaliuyanii* have a total of matured individuals of ca. 5000 plants. The population of the holotype locality is the largest and is in good condition because their occupied area is included in the Nabanhe River Watershed National Nature Reserve and, during our 10 yearly re-visitation, we found the habitats had been well-protected. Therefore, the new species is preliminarily assessed as Least Concern (LC) according to IUCN (2023).

#### Etymology.

The species epithet is named after Prof. Yan Liu, Guangxi Institute of Botany, Guangxi Zhuangzu Autonomous Region and the Chinese Academy of Sciences, who has made great contributions to plant taxonomy in China.

#### Chinese name.

宽翅蛇根草 (kuan-chi-she-gen-cao).

#### Additional specimens examined

**(*paratypes*).** China • Same village as holotype, elevation 1700 m, 2 Apr 2001 (fl.) H. Wang 4311 (PE 2014155!), elevation 1500 m, 7 Jun 2014 (fr.), L. Wu 3706 (BNU! CSFI!), 21°30'42.43"N, 100°30'18.73"E • elevation 1700 m, 13 Apr 2024 (fl.), X.W. Liao LXW0219 (CSFI!), 21°30'27.65"N, 100°30'27.12"E • elevation 1695 m, 13 Apr 2024 (fl.), X.W. Liao LXW0220 (CSFI!).

Vietnam • Dien Bien Province: Muong Nhe Distr., Muong Nhe Natural Reserve, Sin Thau Municipality, Ta Mieu Village, around point 22°24'02"N, 102°08'38"E, elevation 1800–1850 m, old humid secondary evergreen broad-leaved montane forest of very steep mountain slopes composed of sandstone, terrestrial herb to 0.5 m tall, flower pinkish-white, common, 14 May 2022, L. Averyanov, H.T. Tran, K.S. Nguyen, H.C. Nguyen, T. Maisak, C.K. Bac, VR 1637 (HN!, LE!).

#### Notes.

**Figure 2.**
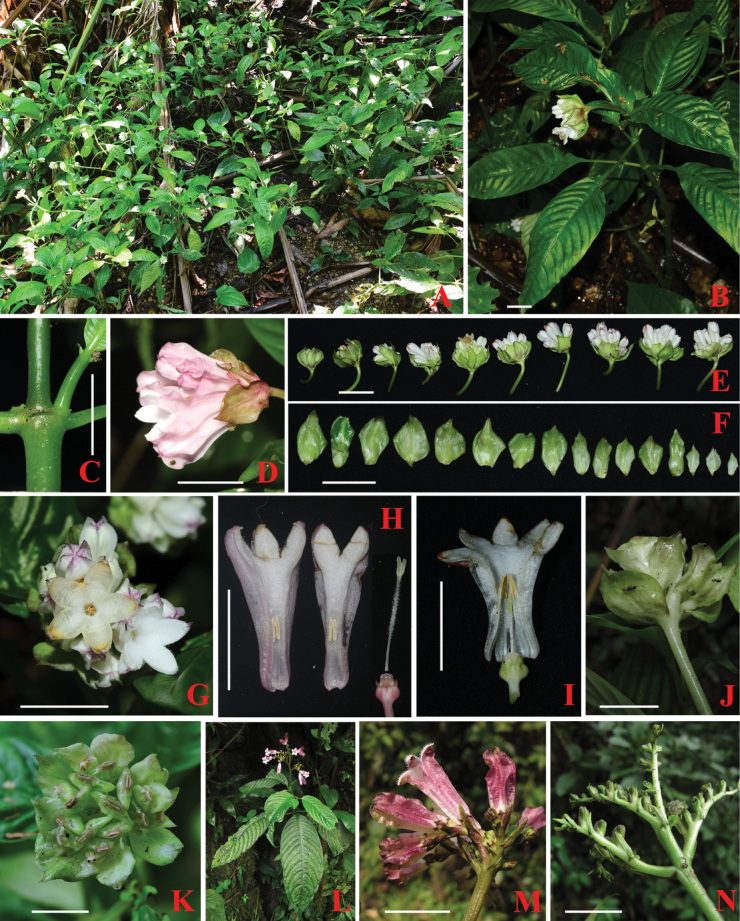
*Ophiorrhizaliuyanii***A** habitat **B** habit **C** stipule **D** inflorescence in side view **E** inflorescences in different development stages **F** bracts from lower part to upper part of inflorescence **G** corollas in top view **H** longitudinally dissected long-styled flower **I** longitudinally dissected short-styled flower **J** infructescence in side view **K** infructescence in top view. *Ophiorrhizaalatiflora***L** habit **M** inflorescence in lateral view **N** infructescence. Scale bars: 1 cm. Photos by L. Wu, X. W. Liao and K. S. Nguyen.

**Figure 3.**
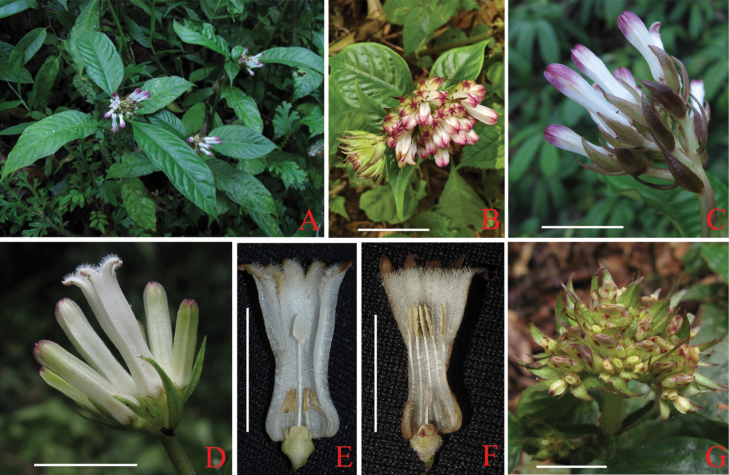
*Ophiorrhizabaviensis***A** habit **B** inflorescence in top view **C, D** inflorescence in side view **E** longitudinally dissected long-styled flower **F** longitudinally dissected short-styled flower **G** infructescence in top view. Scale bars: 1 cm. Photos by L. Wu.

*Ophiorrhizaliuyanii* is morphologically most similar to *O.baviensis* on having congested inflorescences and distinct, persistent bracts. However, the former differs from the latter mainly by its glabrous (vs. pubescent or puberulent) stems, peduncles and calyx (Figs 2B, C, D, E, 3E, F), broad-ovate to ovate (vs. lanceolate) bracts with larger in size, 9–22 × 4–11 (vs. 6–15 × 2–7) mm (Figs 2D–F, 3C, D, G), corolla tubes inside with (vs. without) a ring of white hairs at the middle and anthers positioned near the middle (vs. base) in long-styled flowers (Figs 2H, I, 3E, F). The new species also resembles *O.alatiflora* by having wings longitudinally and wider than 0.8 mm outside corolla, but it clearly differs by its congested (vs. developing) inflorescences and infructescences (Figs 2B, D, E, J, K, M, N), broad-ovate to ovate (vs. linear or linear-lanceolate) bracts, 4–11 (vs. 0.8–1.5) mm wide (Figs 2F, J, K, N). Further distinctive characteristics of the three species are shown in Table 1.

**Table 1.** Morphological comparison of *Ophiorrhizaliuyanii*, *O.alatiflora* and *O.baviensis*.

	***Ophiorrhizaliuyanii***	***O.alatiflora***	***O.baviensis***
stem	glabrous	glabrous	glabrous to densely pubescent
peduncles	glabrous	glabrous	densely pubescent or puberulent
inflorescence	congested	congested when young, then developing when matured	congested
bracts	broad-ovate to ovate, 9–22 × 4–11 mm, apex acute or sometimes obtuse, glabrous	linear or linear-lanceolate, 5–15 × 0.8–1.5 mm, apex acute, glabrous	lanceolate, 6–15 × 2–7 mm, apex accumulate, puberulent or ciliate
calyx	glabrous	puberulent	pubescent, sometimes densely
corolla	subtubular	subtubular	tubular, slightly swollen at base
long-styled flowers inside	inside with a ring of white hairs at the middle	inside with a ring of white hairs at the middle	inside densely pubescent, but without a ring of white hairs at the middle
anthers and stigma	inserted near the middle and the throat of corolla tube in long-styled flowers respectively, while opposite in the short-styled flowers	inserted near the middle and the throat of corolla tube in long-styled flowers respectively, while opposite in the short-styled flowers	inserted near the base and above middle of corolla tube in long-styled flowers respectively, while opposite in the short-styled flowers

## Supplementary Material

XML Treatment for Ophiorrhiza liuyanii

## ﻿Additional information

### Author ORCIDs

Xiao-Wen Liao https://orcid.org/0000-0003-2652-2862

Khang Sinh Nguyen https://orcid.org/0000-0001-5171-4140

Lei Wu https://orcid.org/0000-0003-1451-7855

### Data availability

All of the data that support the findings of this study are available in the main text.
